# Relationship Between Quality of Life, Perceived Stress, and Disease Characteristics in Patients With Ulcerative Colitis in Al-Madinah

**DOI:** 10.7759/cureus.75869

**Published:** 2024-12-17

**Authors:** Sally A Elnawasany, Ahmad Afghani, Abdulaziz M Badarb, Rahaf Almaimani, Zainab Hadram, Ruba Alqahtani

**Affiliations:** 1 Tropical Medicine, Tanta University, Tanta, EGY; 2 Clinical Sciences, Al Rayan College, Madina, SAU; 3 Internal Medicine, King Fahd Hospital, Madina, SAU; 4 Family Medicine, Ministry of Health of Saudi Arabia, Madina, SAU; 5 Internal Medicine, Al Rayan College, Madina, SAU

**Keywords:** disease characteristics, inflammatory bowel diseaes (ibd), perceived stress, quality of life, ulcerative colitis

## Abstract

Background

Ulcerative colitis (UC) is a common chronic disease. Perceived stress is one of the risk factors that stimulate UC activity. Long-term clinical suffering negatively alters the health-related quality of life (HRQOL).

Aim

This study aimed to investigate the relationship between HRQOL, perceived stress, and disease characteristics in patients with UC in Al-Madinah.

Methodology

Between June 2024 and September 2024, a cross-sectional study was conducted on 122 participants. The test group included 61 UC patients who visited the gastroenterology department of King Fahd Hospital, Al Madinah, Kingdom of Saudi Arabia (KSA). Sixty-one healthy volunteers served as controls. Data were collected from the participants through an anonymous questionnaire after their consent. The questionnaire included demographic data, a 10-item Perceived Stress Scale (PSS), a 36-item Short Form (SF-36) Survey, and UC clinical status data from the UC patients.

Results

The mean score of PSS was significantly greater in UC patients (19.23±5.28) than in the control group (11.21±6.644), p < 0.001. Patients with UC suffer significantly (p < 0.05) lower health-related quality of life than the control group. Ulcerative colitis patients experienced the lowest scores in the energy/fatigue (56.15±29.15) and emotional well-being domains (66.69±29.26). Sex standardized (β) (-0.308) and time since diagnosis β (0.336) were good predictors (p < 0.05) of physical functioning. Time since diagnosis β (0.401) and partial Mayo score (p Mayo) score β (-0.353) were good predictors (p < 0.05) of role limitations due to physical health. Time since diagnosis β (0.349) was a good predictor (p < 0.05) of role limitations due to emotional health. For social functioning, sex β (-0.348), smoking β (-0.314), time since diagnosis β (0.421), and extraintestinal manifestations β (-0.260) were good predictors (p < 0.05). Extraintestinal manifestations β (-0.386) were good predictors (p < 0.05) of pain. Time since diagnosis β (0.325) and p Mayo score β (-0.278) were good predictors (p < 0.05) of general health.

Conclusion

Patients with US had lower PSS and HRQOL domains than healthy individuals. Patients with UC experienced the lowest scores in the energy/fatigue and emotional well-being domains. Time since diagnosis, p Mayo score, extraintestinal manifestations, sex, and smoking were good predictors of many domains. This confirms the solidarity of psychological care with medical treatment in those patients.

## Introduction

Ulcerative colitis (UC) is a chronic disease characterized by mucosal hyperimmune inflammation as a response to intra-intestinal stimulus in the presence of genetic predisposition [1]. The clinical presentation of UC varies between periods of remission and exacerbation with subsequent complications and health resource consumption [2]. Patients with UC suffer from abdominal pain, bloody diarrhea, and fatigue, which impact their quality of life (QoL) in the long run [3]. Many risk factors exacerbate UC activity and increase its morbidity, such as aspirin, non-steroidal anti-inflammatory drugs (NSAIDs) [4], and mental stress [5].

Perceived stress is a common worldwide problem. It is defined as a subjective emotion or idea about the burden on the individual for a certain duration or a fear of losing the ability to control life [6]. It is not related to the number of stressful events but depends on how the person thinks about his success in overcoming stress [6].

Depressive mood badly affects health-related quality of life (HRQOL), which in turn complicates the course of UC. Many studies investigated the relationship between stress and QoL and their impact on disease severity and course in UC patients. Popa et al. (2022) explored the impact of inflammatory bowel diseases on perceived stress and quality of life. They reported a significant correlation between higher levels of stress and increased inflammatory bowel disease (IBD) activity, which has prompted the development of problem-focused coping mechanisms [3]. Another study postulated that the progression of IBD is negatively impacted by psychological variables such as anxiety-related depression and low HRQOL [7]. Patients with ulcerative colon illness experience symptoms for extended periods, which has a detrimental impact on their social, functional, and financial lives. When faced with an active illness, patients' QoL is lower than that of patients with a quiescent illness [3]. This highlights the importance of assessing and improving perceived stress and HRQOL as a routine strategy in UC patients' management plans [7]. This study aims to investigate the relationship between HRQOL, perceived stress, and disease characteristics in patients with UC in Al-Madinah.

## Materials and methods

Between June 2024 and September 2024, a cross-sectional study was conducted on 122 participants. Sixty-one UC patients consecutively visited the gastroenterology department of King Fahd Hospital, Al Madinah, Kingdom of Saudi Arabia (KSA), and 61 healthy individuals as control (patients' relatives, medical students, and medical trainees in King Fahd Hospital) were included in the study. The control group included people demographically matching the study group without any medical issues or psychological problems. Ulcerative colitis diagnosis was based on endoscopic examination and biopsy. Patients who refused to share in the study or who had any mental illnesses or psychopharmacological treatment/psychotherapy, pregnancy, or other chronic diseases were excluded. 

Data collection

Data were collected from the participants who consecutively visited the hospital during the study period. through an anonymous questionnaire after their consent. This questionnaire included demographic information such as age, sex, occupational and marital status, living environment, and level of education, as well as personal and familial medical history.

Patients’ subjective perception of stress

The Perceived Stress Scale (PSS), developed by Cohen et al. in 1983, was used to assess patients’ subjective perception of stress related to symptoms experienced over the past month [8]. The PSS is a 10-item tool designed to measure the degree to which situations in one’s life are appraised as stressful. Responses are recorded on a five-point Likert scale ranging from 0 (never) to four (very often) [8]. The reliability and validity of the original scale were established by Cohen and Williamson, with a Cronbach’s alpha of 0.75 observed in this study [9]. The Arabic translation of the scale, validated in previous research, was utilized for this study [10].

Health-related quality of life

Health-related quality of life was assessed using the Short Form-36 (SF-36) Survey, a tool developed by Ware and Sherbourne in 1992 to evaluate health status across eight domains [11]. Reliability and validity of the original scale have been well established, with internal consistency reported to be strong (Cronbach’s alpha > 0.70 across domains [11]). Scoring was based on a 0-100 scale for each domain, where higher scores indicate better health-related quality of life. The SF-36 contains 36 items distributed across the following categories: physical functioning (10 items), role limitations due to physical health problems (role physical, four items), role limitations due to emotional health problems (role emotional, three items), energy/fatigue (four items), emotional well-being (five items), social functioning (two items), pain (two items), general health (five items), and one item assessing overall general health perception. The Arabic translation of the SF-36, validated in previous studies, was used in this research [12].

Clinical status of UC

Clinical status data such as disease activity by using a partial Mayo score (p Mayo) [13], the time that passed after the first diagnosis, extraintestinal manifestations (arthritis, aphthous ulcers, uveitis), current and previous medication (medication history of more than one month), ulcerative colitis-related surgical history, and family history for IBD. These data were confirmed from the medical records of each patient. The questionnaires of the control group included socio-demographic data, PSS, and HRQOL.

Statistical analysis

Data was analyzed using IBM SPSS Statistics for Windows version 20 (IBM Corp., Armonk, NY). For descriptive analysis, data were expressed as frequency and percentage. Chi-square and Fisher’s exact tests were conducted to compare the categorical variables. The predictive model for quality-of-life domains was assessed using analysis of covariance (ANCOVA) and linear regression analysis to demonstrate (R2) of the linear model and its standardized (β) coefficient. The formula used for prediction was Y = b0 + b1, where b0 is the Y-intercept and b1 is the slope. All tests determined significance at a two-tailed p-value ≤ 0.05.

## Results

Regarding demographic data, the mean age in the study group was 36.26±14.86 years, and 30.26±12.22 years in the healthy control group (p <0.05). Females constituted 59.02% (36) of the study group and 50.82% (31) of the control group (p > 0.05). Thirty-seven (60.7%) participants in both groups were single. Of the UC patients, 41 (67.2%) were unemployed, and 40 (65.6%) were unemployed in the healthy individuals group (p > 0.05). Thirty-five (41.7%) UC patients and 19 (31.1%) in the control group (p > 0.05) were highly educated. There is a significant difference in the living environment between both groups (p < 0.05); independent living was observed in 19 (31.1%) UC patients and seven (11.5%) healthy individuals (Table 1).

**Table 1:** Demographic criteria in patient and control group *P < 0.05

		Study	Control	t/x^2^	p
Age		36.26±14.86	30.26±12.22	-2.436	0.016*
Gender	Male	25 (40.95%)	30(49.18%)	0.828	0.363
	Female	36 (59.02%)	31(50.82%)		
Marital status	Single	37(60.7%)	37(60.7%)	1.333	0.721
	Married	22(36.1%)	22(36.1%)		
	Widow	1(1.6)	0 (0.0%)		
	Divorced	1(1.6%)	2 (3.3%)		
Occupation status	Unemployed	41(67.2%)	40 (65.6%)	0.037	0.848
	Employed	20(32.8%)	21(34.4%)		
Education level	Not educated	6 (10.0%)	13(21.3%)	4.351	0.226
	High school	11 (18.3%)	16 (26.2%)		
	Beyond secondary school	18 (30.0%)	13 (21.3%)		
	Higher education	25 (41.7%)	19 (31.1%)		
Living environment	Independent	19 (31.1%)	7 (11.5%)	7.038	0.008*
	With family	42 (68.9%)	(88.5%)		

Regarding the clinical criteria of UC patients, the mean p Mayo score was 3.20±2.70. Remission was reported in 22 (36.07%) patients, while 16 (26.23%) were with mild activity, 17 (27.87%) were with moderate activity, and six (9.84%) were with severe activity. Extraintestinal manifestations were detected in 19 (31.15%). Most patients, 29 (47.54%), were diagnosed with UC for a period of less than one year. Twenty-two (36.07%) of patients were treated with biological therapy. Neither family history of UC nor UC-related history was detected (Table 2).

**Table 2:** Clinical criteria for ulcerative colitis patients

Ulcerative colitis data	No. (%)
Remission	22 (36.07%)
Mild activity	16 (26.23%)
Moderate activity	17 (27.87%)
Sever activity	6 (9.84%)
Mean p Mayo score	3.20±2.70
Extraintestinal manifestations	19 (31.15 %)
Duration since diagnosis: <1 year	29 (47.54%)
Duration since diagnosis: 1-5 years	10 (16.39%)
Duration since diagnosis: >5 years	22 (36.07%)
Biological therapy	22 (36.07%)
Other therapies	39 (63.93 %)
Family history of inflammatory bowel disease (IBD)	0 (0%)
Surgical history	0 (0%)

Regarding PSS, UC patients with moderate perceived stress were 25 (41%); in the control group, 46 (75.41%) of the healthy participants showed mild PSS. The mean score of PSS was significantly greater in patients (19.23±5.28) than the control group (11.21±6.644), p <0.001 (Table 3).

**Table 3:** Perceived Stress Scale (PSS) in ulcerative colitis patients and healthy individuals *** p<0.001

PSS	Study	Control	x^2^	P
Mild	35 (57.4%)	46 (75.41%)	4.596	0.100
Moderate	25 (41.0%)	14 (23.0%)		
Severe	1 (1.6%)	1 (1.6%)		
			t	
Mean score ±SD	19.23±5.28	11.21±6.64	-7.376	0.000***

We compared the PSS mean score in patients with mild, moderate, and high UC activity. Notwithstanding, higher PSS mean scores were observed in patients with high UC activity. There was no significant correlation between PSS and p Mayo correlation coefficient (0.003), p > 0.05 (Table 4).

**Table 4:** Perceived Stress Scale (PSS) in ulcerative colitis (UC) patients and healthy individuals

	Mild UC activity	Moderate UC activity	High UC activity	F	p
PSS means	19.94±5.36	18.35± 6.07	20.6667 ± 1.51	0.511	0.604

Health-related quality of life was measured in UC patients and controls, where a high score represents a better quality of life. The UC patients had lower health-related quality of life scores than the control group. In UC patients, the lowest scores were observed in the energy/fatigue (56.15±29.15) and emotional well-being domains (66.69±29.26). In healthy individuals, lower scores were detected in the energy/fatigue (65.49±19.49) and general health (72.56±17.18) domains (Table 5).

**Table 5:** Health-related quality of life (HRQOL) domains in UC patients and healthy individuals * P < 0.05, *** p<0.001 SF-36: Short Form-36 Survey

SF-36 domains	Domains	Study	Control	t	P
	Physical functioning	81.31±20.95	82.80±18.87	0.461	0.646
	Role limitations due to physical health	74.59±39.39	87.70±19.70	2.253	0.026*
	Emotional well being	66.69±29.26	76.46±16.08	2.295	0.023*
	Energy/fatigue	56.15±29.15	65.49±19.49	2.134	0.035*
	Role limitations due to emotional problems	73.22±40.74	77.05±42.40	0.595	0.553
	Social	80.74±23.56	82.09±16.98	0.365	0.716
	Pain	75.78±27.59	91.84±12.39	4.148	0.000***
	General health	70.69±17.84	72.56±17.18	4.148	0.000***

ANCOVA was used to detect the determining factors of HRQOL in UC patients regarding the predictive model for the effect of different clinical parameters (sex, smoking, time since diagnosis, extraintestinal manifestations, and treatment) as predictors of QoL domains. Physical functioning, role limitations due to physical health, role limitations due to emotional problems, social functioning, pain, and general health were significant fit models. In the physical domain, sex (being female correlates with lower scores in this domain) and time since diagnosis were significant independent predictors. The role of the physical domain, time since diagnosis, and p Mayo score were significant independent predictors. Regarding role emotional, time since diagnosis was the only significant independent predictor. The social domain was independently predicted by sex and smoking, time since diagnosis, and the presence of extraintestinal manifestations. The pain domain had only a single independent predictor: the presence of extraintestinal manifestations. The general health domain has had time since diagnosis and p Mayo as an independent predictor (Table 6).

**Table 6:** The predictive value of the health-related quality of life domains over clinical factors

Goodness of fit	Physical functioning	Role limitations due to physical health	Role limitations due to emotional health	Energy/fatigue	Emotional well being	Social functioning	Pain	General Health
R^2^	0.252	0.322	0.226	0.094	0.175	0.306	0.224	0.220
P value	0.01	0.001	0.026	0.479	0.096	0.002	0.028	0.031
Standardized coefficient								
Sex	-0.308	-0.040	-0.258	-0.317	-0.276	-0.348	-0.129	-0.205
Smoking	0.007	-0.086	-0.117	-0.158	-0.039	-0.314	0.013	0.110
Time since diagnosis	0.336	0.401	0.349	0.073	0.214	0.421	0.181	0.325
Extraintestinal manifestations	-0.038	-0.177	-0.045	-0.095	-0.021	-0.260	-0.386	-0.106
P Mayo	-0.205	-0.353	-0.242	0.006	-0.108	0.014	-0.190	-0.278
Treatment	-0.174	-0.131	-0.164	-0.114	-0.225	0.137	-0.049	0.045
p for model parameters								
Sex	0.037	0.769	0.084	0.051	0.074	0.015	0.383	0.170
Smoking	0.963	0.548	0.444	0.340	0.803	0.033	0.930	0.474
Time since diagnosis	0.009	0.001	0.008	0.599	0.108	0.001	0.160	0.014
Extraintestinal manifestations	0.755	0.13	0.714	0.477	0.870	0.029	0.003	0.390
P Mayo	0.106	0.004	0.062	0.963	0.411	0.909	0.141	0.033
Treatment	0.242	0.272	0.201	0.409	0.091	0.259	0.700	0.724

The prediction formulae are as follows:

Physical=84.73+(sex×−11.086)−(smoking×0.333)+(Time since diagnosis×8.093)+(extraintestinal manifestations×−1.440)+(Mayo score×−1.350)+(treatment×−6.550)

Role Physical=91.033+(sex×−2.249)−(smoking×−6.330)+(Time since diagnosis×14.922)+(extraintestinal manifestations×−10.482)+(Mayo score×−3.610)+(treatment×−9.045)

Role Emotional=106.453+(sex×−12.267)−(smoking×−7.371)+(Time since diagnosis×11.081)+(extraintestinal manifestations×−2.268)+(Mayo score×−2.107)+(treatment×−9.638)

Energy=122.183+(sex×−18.681)−(smoking×−12.380)+(Time since diagnosis×2.872)+(extraintestinal manifestations×−5.929)+(Mayo score×0.069)+(treatment×−8.310)

Emotional=93.236+(sex×−16.54)−(smoking×−3.122)+(Time since diagnosis×8.610)+(extraintestinal manifestations×−1.323)+(Mayo score×−1.194)+(treatment×−16.669)

Social=121.679+(sex×−28.556)−(smoking×−34.299)+(Time since diagnosis×23.139)+(extraintestinal manifestations×−22.675)+(Mayo score×0.209)+(treatment×−13.899)

Pain=126.069+(sex×−10.257)−(smoking×1.423)+(Time since diagnosis×9.631)+(extraintestinal manifestations×−32.581)+(Mayo score×−2.767)+(treatment×−4.827)

General=78.671+(sex×−8.662)−(smoking×6.164)+(Time since diagnosis×9.181)+(extraintestinal manifestations×−4.767)+(Mayo score×−2.158)+(treatment×2.358)

## Discussion

The burden of IBD is a worldwide problem [14] where the chronicity of the disease negatively impacts the patient's QoL [15]. At the same time, stress affects the clinical course of UC by triggering disease exacerbation [16]. 

This study aims to assess the QoL and the perceived stress of ulcerative colitis patients and to find possible influencing factors affecting their QoL domains. The mean age of UC patients was about 36 years, which aligns with the global UC age average [17].

The PSS reflects the individual’s perception of stress; it has three categories: mild, moderate, and severe. In our study, UC patients had a significantly higher PSS mean than the control group. These findings follow the results of another Romanian cross-sectional study on 70 IBD patients and 70 healthy controls, where the prevalence of high stress and greater mean scores was detected in IBD patients [3]. 

However, in our study, patients with severe UC activity had larger PSS mean scores; there was no significant correlation between PSS and p Mayo. It has been proven that patients' feelings of stress depend more on their perception of performance deficit than on the severity of UC [18]. This emphasizes the importance of focusing more on the patient's sense of functional incapacity and not only on the severity of the condition [15]. High-stress perception raises the chance of symptoms relapse [5,19]. The hypothalamic-pituitary-adrenal (HPA) axis and the parasympathetic and sympathetic branches of the autonomic nervous system (ANS) are the main participants in the stress response system, and gut-brain axis disturbance [20].

Inflammatory bowel disease has a major effect on patient QoL. Some IBD symptoms, like diarrhea, abdominal pain, fecal incontinence, and rectal bleeding, can be emotionally and physically taxing [21]. Ongoing monitoring and adaptation by patients and family members are necessary for IBD. Psychosocial discomfort seriously impairs the patient's QoL and ability to manage themselves [22]. Both physiological and medical treatment achieve effective IBD management to improve the quality of patients' lives [21].

We evaluated HRQOL through the SF-36 questionnaire. Improved mental and physical health is shown by higher ratings. All domains of HRQOL were lower in UC patients compared to the healthy control. Similar results were reported in other studies [3,23].

We tried to discover the factors that alter HRQOL in UC. In this study, time since diagnosis showed a significant positive correlation with physical functioning, role limitations due to physical health, role limitations due to emotional problems, social functioning, and general health domains. This result was confirmed by numerous studies that demonstrated that the HRQOL of IBD patients improved over time, declining during the time of the diagnosis and then significantly improving afterward [24-26]. In a European cohort study, 1,560 patients with IBD from 31 centers participated. The patients answered QoL questionnaires at diagnosis and after one year. QoL was improved after one year [25]. McCombie et al. (2015) reported similar findings in their study when they measured the improvement of QoL after six months of the diagnosis in IBD patients [26]. On the other hand, Popa et al. (2022) couldn’t find any correlation between any factors and the HRQL of IBD patients in their cross-sectional study of 70 IBD patients [3].

In our study, we detected a significant negative correlation between the p Mayo score and role limitations due to physical health and general health domains.

This is in accordance with an Italian study where patients with increased activity reported more malaise, weakness, pain, and sleep interruption. highlighting that severe UC makes worse health quality in patients [27].

Poor QoL was observed in patients with extraintestinal manifestations. [28]. This was confirmed in this study, where we observed that extraintestinal manifestations predict low social functioning and pain domains. These findings are similar to the results of a cross-sectional study on 201 patients to find factors affecting UC patients' QoL. The existence of extraintestinal manifestations was found to be an independent factor of UC disability [28]. Chronic symptoms of UC make the patient feel stigmatized in society and badly affect his social communications [29].

Moreover, smoking significantly negatively correlated with the study patients' social functioning domain. It was postulated that there is a negative correlation between smoking and QoL, and the strength of this correlation varies with cigarette consumption. Additionally, quitting smoking greatly enhances the QoL [30].

Treatment of IBD enhances the QoL, mainly Crohn’s disease, while lower quality of life was noticed in UC patients who are under conventional therapy [15,25].

We did not find a correlation between treatment and improvement of HRQOL domains. It may be explained by the inability of patients to feel a discernible improvement in their QoL during the treatment period or by suffering from the medication side effects.

Although this study highlighted the high perceived stress and impairment of HRQOL in UC patients and detected significant influencing factors of several QoL domains, it was limited by its short duration. Prolonged time will ensure higher accuracy and help with detailed analysis. Using specified QoL assessment tools for IBD and UC could cover more aspects of patients' suffering, such as sexual functioning, low fertility, and self-esteem. These limitations can be considered in future research.

## Conclusions

This work sheds light on UC patients' psychological and life aspects, which have a bidirectional relationship. Impaired QoL is associated with social and economic burdens on the patients and their families, which adds to their misery. A comprehensive framework for managing UC that encompasses behavioral, emotional, and cognitive aspects in addition to medical and psychological care has been promoted because patients with active UC have a clinically significant burden of disease across the majority of QoL domains. Since improving QoL is a primary goal of therapy, its assessment is a crucial component.
